# The Impact of Addition Oats *(Avena sativa)* and Cinnamon on Cookies and their Biological Effects on Rats Treated with Cirrhosis by CCL4

**DOI:** 10.1016/j.sjbs.2021.08.010

**Published:** 2021-08-09

**Authors:** Ahmed A. Aly, Eid A. Zaky, Hadeer A. Mahmoud, Abdulmajeed F. Alrefaei, Ahmed M. Hameed, Hussain Alessa, Abdulrahman A. Alsimaree, Mohammed Aljohani, Salah M. El-Bahy, Sultan Kadasah

**Affiliations:** a Home Economics Department, Faculty of Specific Education, Benha University, Egypt; b Department of Biology, Jamoum University College, Umm Al-Qura University, 21955 Makkah, Saudi Arabia; c Department of Chemistry, Faculty of Applied Sciences, Umm Al-Qura University, 21955 Makkah, Saudi Arabia; d Department of Basic Science (Chemistry), College of Science and Humanities, Shaqra University, Afif, P.O. BOX 33, Shaqra 11961, Saudi Arabia; e Department of Chemistry, College of Science, Taif University, P.O. Box 11099, Taif 21944, Saudi Arabia; f Department of Chemistry, Turabah University College, Taif University, P.O. Box 11099, Taif 21944, Saudi Arabia; g Department of Biology, Faculty of Science, Bisha University, Saudi Arabia

**Keywords:** Oats (*Avena sativa*), Cinnamon, Chemical composition, Sensory evaluation, Microbiological evaluation, Biological assays, Histopathological

## Abstract

Oats are represented an important source for nutrition, and it have attracted a lot of attention in recent years. In this study we produced oatmeal cookies and oats was added to formula wheat by 50% and 75%. Also, we added cinnamon to treatments 3 and 4 by 5% to increase nutrition value of oatmeal cookies, improvement sensory evaluation and increase antioxidant properties. All the cookies treatments were storage at room temperature. The purposes of this study were to study antioxidant activity for oats and cinnamon. Also determined the chemical composition include (moisture content, protein, ash, total lipids, crude fibers, carbohydrates and total calories) for oats, white flour and oatmeal cookies treatments. In addition evaluated all of sensory evaluation contain (appearance, color, texture, taste and odor) and microbiological evaluation such as (total bacterial count, spore forming bacteria and mold and yeasts count) for oatmeal cookies treatments. Also, biological assays were preformed to measure level of GPT, GOT, urea, creatinine, total cholesterol, triglycerides, HDL, LDL, and glucose. Furthermore, histopathological examination of both liver and kidneys was analyzed. The obtained results were clarified that the antioxidant activity for oats and cinnamon were 52.91% and 87.91%. Moreover, addition of oats and cinnamon to the cookies improve sensory evaluation as made it more acceptable, decreasing microbial load principally treatments 4 (prepared by 75% oats + 5% cinnamon). Biochemical assays were improved in rats with cirrhosis of carbon tetrachloride and their histopathological examination of liver and kidneys. It was clear that the additions ground whole grain oats and cinnamon to wheat flour based cookies improve its nutritional, chemical, and biological functions properties.

## Graphical abstract

**Figure:**
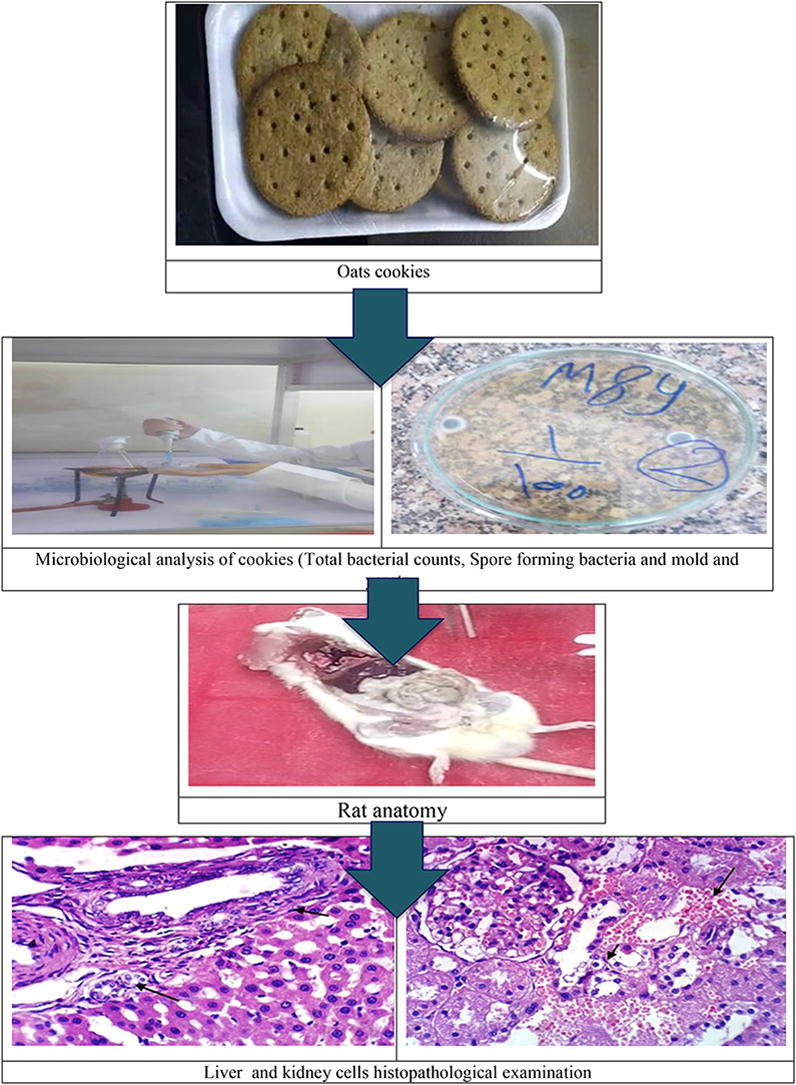

## 1 Introduction

*Avena sativa*, (popular oats) belongs to the poaceae family and the genus *Avena*, that include about 70 sorts, but a little of this are cultivated. Oats *(Avena sativa*) considered the most important kind among cultivated oats (*A.orientalis*, *A.sativa, A.byzantina, A. diffusa,* among other) and that due to their multifunctional and nutritional properties. Oats a cool and minor season crop which have been used as a foodstuff for Scandinavian, Ireland, and the Scotland countries. Oats considered one of the most important crops all over the world. It becames in the six rinks for grains both of livestock and human in Poland, Finland, Millennia, Russia, Canada, and US are leading oats products countries. The fifth place of the oats yields in all the world goes to Poland. It is the main crop in Germany after sorghum, maize, wheat, rice, and barley (Petkov et al., 2001, Suttie and Reynolds, 2004, Butt et al., 2008, Gazal et al., 2014).

Oats are normally considered healthy, which was being promoted commercially as a nutritious that has leads to the more wide appreciation of oats such as food for humans. Oats have the largest amount of protein compared to the other cereal grains as well as oats represent to be an excellent and that may be due to its ability to composition the essential amino acids in an adequate amount (Mohamed et al., 2009). Oats have been intake for long centuries as a nutrition crop because of its containing of balanced protein, vitamin B and E, high dietary fibers, unsaturated fatty acids in addition to bioactive composition like polyphenols, sterols, beta glucans, avenanthramides and phenolic (Peterson et al., 2002, Collins, 2011, Gangopadhyay et al., 2015). The starch of oats is used in many products like, cardboard, brown paper products as well as fat replacers coating agents for tablet formularization in addition to cleanser and cosmetic products (Autio and Eliasson, 2009, Zwer, 2016). Antioxidants of oats contain many important industrial as it retaining the texture, taste, flavor, slowing the lipid oxidation in addition to that their nutritional content (Sindhi et al., 2013).

There are many studies that indicate that eating whole grains oats effected on ascardiovascular disease (CVD), certain cancers and type2 diabetes (Reynolds et al., 2019). Also whole grain oats caused many effects as like reduce the ratio the level of blood glucose as well as the insulin requires per day for diabetic patients (Storz & Helle, 2019). EA fraction had a high content of antioxidant activity and phenolic between all the fractions (Cai et al., 2011, Tian et al., 2012). Pre-treatment with EA fraction which contains avenanthramides made a decrease in the levels of liver enzymes in blood serum in addition to facilitated in the prevention of alcoholic liver injury (Hassanein & El-Amir, 2017).

The main type of cinnamon is called Cinnamomum cassia Presl which widely grown in Yunnan, Hainan, Guizhou Provinces in China, Guangdong and Guangxi. As well as it's also distributed in Seychelles, Madagascar, Vietnam, Sri Lanka and India (He et al., 2016). Cinnamon (Cinnamomum) is the genus from the Lauraceae family that has hundreds of species in all the world moreover 4 types of them considered the main use in commercial applications. The main kinds of cinnamon are Cassia cinnamon, Cinnamomum zeylanicum, Cinnamomum loureiroi and Cinnamomum burmanni Java and those kinds are very similar with each other except a little differences in color, shape and taste (Kawatra and Rajagopalan, 2015, Reygaert, 2017).

In the past cinnamon used as dietary herbal medicine as well as it has many properties such as anticancer, anti-inflammatory, antimicrobial, lipid lowering, and antioxidant activity (Gruenwald et al., 2010, Rao and Gan, 2014). The major components in cinnamon are eugenol, cinnamic acid, coumarin, and cinnamaldehyde that have roles in many biological activities like anti-microbial, antioxidant, antifungal, anti-diabetic and anti-inflammatory activities (Zaidi et al., 2015).

Cinnamon at present is considered one of the markets like a prophylactic supplement for insulin resistance, for metabolic syndrome, hyperlipidemia and arthritis (Rafehi et al., 2012, Medagama, 2015). Supplementation which contains cinnamon may significantly decrease all of DBP and SBP in people with type 2 diabetes (Akilen et al., 2013a, Akilen et al., 2013b, Sengsuk et al., 2016, Jamali et al., 2020). Intake cinnamon prevents biosynthesis as well as esterification all of triacylglycerol and cholesterol in healthy rats (Lopes et al., 2015).

In French herbal book called “Le Livre des Simples Medecines” wrote in a 15th-century, which contain cinnamon and other six medicinal spices (mace, pepper, ginger, cinnamon, cloves and nutmeg). The text states that cinnamon can be used for weakness of the liver, stomach as well as digestion 'for restore appetite’ also recently it used for heart ailments, syncope, cracked lips and other sores (Nam, 2014).

Cinnamon supplementation may be affect blood pressure as improve it modestly(Hadi et al., 2020). Cinnamon antioxidant probably reduction the endothelial dysfunction and therefore it due to a lower in blood pressure (Akilen et al., 2013a, Akilen et al., 2013b).

Liver cirrhosis is considered the third stage of liver caused by frequent consequences of all chronic diseases (CLD) differentiates by reiterated parenchymal damage. Cirrhosis is differentiating by diffusion process of tissue fibrosis and conversion of the normal architecture of the liver into structurally abnormal nodules (Anthony et al., 1978). Cirrhosis and chronic liver failure are the major reasons for mortality and morbidity, as the plurality of preventable conditions referred to excessive alcohol consumption or nonalcoholic fatty liver disease, or viral hepatitis. Cirrhosis usually is an inactive disease; many patients rest without visible symptoms pending the variceal bleeding from portal hypertension, the occurrence of decompensation, characterized by hepatic encephalopathy, ascites or spontaneous bacterial peritonitis. Physical examination for patients with cirrhosis can detect many results, which necessitate a gastrointestinal-or hepatic-based work up determining the etiology (Heidelbaugh and Bruderly, 2006). The aim of this investigation is 1) to make a composition between whole grain oats and wheat flour and production cookies with distinct condensations of oats that rich in dietary fiber, antioxidant activity, protein, fat, minerals and vitamins. 2) to study the chemical composition and microbiological evaluation of the cookies. 3) to analyze the impact of cookies supplemented with oats and cinnamon on rats with cirrhosis.

## 2 Materials and method

### 2.1 Materials

Oats, wheat flour, and cinnamon, were obtained from the local market in Benha, Kalyobiya governorate, Egypt. Chemicals that used in chemical, microbiological analysis were obtained from El-Gomohoria for chemical industries company, Cairo, Egypt. 10% liquid solution of carbon tetrachloride CCl_4_ was used to poisoning the liver according to (Passmore & Eastwood, 1986**)**. Meanwhile olive oil was acquired from the local pharmacy to use in loosening pending the induction.

#### 2.1.1 Preparation of cookies

Four formulas cookies (Supplemented with 50 % oats, 50% oats + 5% cinnamon, and 75% oats + 5% cinnamon) were prepared according to the method described by (McMinn et al., 2007). The treatments kept at room temperature (25 ± 2) during storage period (three months).

### 2.2 Methods of analysis

#### 2.2.1 Chemical analysis

Moisture content, ash content, crude protein, total lipids and crude fibers were determined according to the methods described by (AOAC, 2005). Carbohydrates of prepared cookies formulas were calculated by the difference as the following equation: Total Carbohydrates% = 100 - %(Protein + Fat + Ash + Fibers). Results calculated as (g/100 g on dry weight basis). Total calories Energy value was estimated by the following equation according to (AOAC, 2005): Energy value = (Carbohydrates × 4.0 + Protein × 4.0 + Fat × 9.0).

#### 2.2.2 DDPH radical scavenging activity

The antioxidant activity of oats, and cinnamon were estimated by using the stable 2.2-dipheny1-1-picry1-hydrazy1 radical (DPPH) according to the methods of (Aly et al., 2020).

#### 2.2.3 Organoleptic tests

Organoleptic evaluation i.e appearance, color, texture, taste and odor of the prepared cookies formulas by using oats, and cinnamon were evaluated by the panelists of stuff members of Home Economics, Faculty of Specific Education, Benha University. The sensory evaluation was performed 24 h after baking and during the storage period (3 months) described by (Aly et al., 2021a).

#### 2.2.4 Microbiological analysis

The microbiological evaluation of the prepared cookies formulas were carried the following: total bacterial count, spore forming bacteria and mold and yeasts according to (Aly et al., 2020).

#### 2.2.5 Biological evaluation

##### 2.2.5.1 Rats and housed

Twenty male albino rats weight between (150–160 g) were used in this study. The animals were obtained from Agriculture Research Center, Giza, Egypt. Rats were housed in cages made from a wire under the ordinary laboratory condition and fed on a basal diet for 7 consecutive days as adaptation period. Diets were introduced to rats in a special non-scattering feeding cup to shun feed loss and contamination. Tap water provided to rats via glass tubes protruding out of wire cages from inverted bottles supported in the cages from one side.

##### 2.2.5.2 Induction of liver intoxication in rats

Fifteen male albino rats were administrated by intraperitoneal injection of carbon tetrachloride (CCl_4_) in olive oil 5 % V/V (2 ml/ kg B. wt.) twice a week for the two weeks to induce chronic liver damage according to the method described by (Jayasekhar et al., 1997).

##### 2.2.5.3 Experimental designs and animal groups

The experimental was conducted in the Agricultural Research Center, Giza, Cairo. Rats were housed in cages made from a wire at a room temperature (25C°) and kept under ordinary hygienic conditions. All the rats were fed a standard diet for 7 consecutive days as an adaptation period. Then, rats were distributed into 4 groups each of (5) rats in which means of rat's weight for all groups were nearly equal. All the groups of all experimental were according to the following:

Group 1: Control negative group (-ve), in which normal rats were fed on basal diet and tap water.

Group 2: Control positive group (+ve), in which rats inflicted with hepatotoxicity by CCl_4_were fed on basal diet and tap water.

Group 3: Rats with impaired liver, treated with cookies without oats.

Group 4: Rats with impaired liver treated with oats cookies (prepared with 75% oats and 5% cinnamon). The period of experiment occurred for 28 days.

##### 2.2.5.4 Blood samples and organs

From all the previously mentioned groups, at the end of the experiment blood sample were collected after 12 h of fasting. Using the *retro*-orbital method, by means of a microcapillary glass, blood was collected into a dry clean centrifuge tube, and left it for half one hour to clot at room temperature. To separate serum the blood was centrifuged at 3000 r.p.m. for 10 min. Serum was painstakingly aspirated and put into clean quit fit tubes made from plastic and kept the temperature at −20 °C till the analysis time. The organs liver, kidney, were removed then washed in saline solution and stored in 10% formalin solution according to methods described by (Aly et al., 2021b).

##### 2.2.5.5 Biological analysis

Glutamic pyruvic transaminase determination GPT, Glutamic oxalo acetic transaminase determination GOT, triglycerides, total cholesterol, LDL (low density lipoproteins), HDL (high density lipoproteins), creatinine, serum uric acid and serum glucose were determined according to the method described by (Aly et al., 2021b).

#### 2.2.6 Histopathological examination

Specimens of the internal organs (liver and kidney) were taken straightway after the rats are sacrificed and submerged in 10% formalin neutral stock. After that the fixed specimens were trimmed and dehydrated in ascending grades of alcohol, cleared, in xylene, embedded in paraffin, sectioned (4–6 Mm thickness), stained with hematoxylin and eosin and examined microscopically (Duan et al., 2020).

#### 2.2.7 Statistical analysis

Statistical analysis all the data were expressed as means ± stander deviations (SD) of three replicates. Statistical calculations were executed using method described by (Ismail et al., 2020).

## 3 Results

### 3.1 Chemical compositions

#### 3.1.1 Gross chemical composition of wheat flour and oats flour samples (on a dry weight basis)

Table 1 shows comparison of the chemical composition both of oats flour and wheat flour samples. The moisture content in the wheat flour sample was higher than the whole oats flour sample. As the ratio of moisture in the wheat flour was 10.62%, meanwhile the whole oats flour had 9.69 %. Total lipids in wheat flour were lower than which whole oats flour and the ratios were 1.76% for wheat flour and 5.44 % for whole oats flour. Ash content was 0.87 % in wheat flour and 2.10 % in whole oats flour and that may be due to the whole oats flour ingredients. Whole oats flour recorded higher score in protein (12.64%) than wheat flour sample 10.30 %. Fiber content in the samples were 2.03 % and 3.24 % as whole oats flour sample recorded the higher score compared to wheat flour sample. Total carbohydrates of wheat flour sample were the highest (74.42%) meanwhile; the whole oats flour was the lowest (66.89%).

**Table 1:** Gross chemical composition of whole oats flour and wheat flour samples (on dry weight basis).

Constituents (%)	Sample		L.S.D*0.05
	Whole oats flour	Wheat flour	
Moisture	9.69 ± 0.29^b^	10.62 ± 0.30^a^	0.67
Total lipids	5.44 ± 0.34^a^	1.76 ± 0.22^b^	0.65
Ash	2.10 ± 0.17^a^	0.87 ± 0.16^b^	0.37
Crud protein	12.64 ± 0.35^a^	10.30 ± 0.35^b^	0.69
Fiber	3.24 ± 0.20^a^	2.03 ± 0.13^b^	0.38
Total carbohydrate	66.89 ± 0.87^b^	74.42 ± 0.47^a^	1.58

Mean ± SD with the same latter in the same column are not significantly different (P ≤ 0.05) *: Least significant differences.

#### 3.1.2 Gross chemical composition of oats cookies samples (on a dry weight basis)

Table 2 shows the chemical composition of oats cookies treatments. The ratio of moisture ranged between 3.07% and 3.52% the highest content of moisture found in cookies treatments supplemented with 50 % oats while the lowest one was in cookies treatment supplemented with 75% oats + 5% cinnamon. Total lipids ranged between 16.78% and 18.65% as the control cookies treatment recorded the lowest score; on the other hand in cookies treatment supplemented with 75% oats + 5% cinnamon recorded the highest. Ash content was ranged between 1.83 % and 2.65%, and the cookies treatment supplemented with 75% oats + 5% cinnamon had the highest result and that probably goes to its ingredients. Cookies treatment supplemented with 75% oats + 5% cinnamon contains the highest score of protein (8.52 %) but control cookies treatment was the lowest ratio of protein (5.83 %). Fiber content in cookies treatment supplemented with 75% oats + 5% cinnamon contains the highest score as its ratio 3.24% but control cookies treatment recorded the last score. Total carbohydrates were ranged from 63.88% to 72.38% as the control cookies treatment was the highest meanwhile the lowest observed in cookies treatment supplemented with 75% oats + 5% cinnamon.

**Table 2:** Gross chemical composition of cookies samples (on dry weight basis).

Constituents (%)	Cookies treatments				L.S.D*0.05
	Control	Supplemented with 50 % oats	Supplemented with 50% oats + 5% cinnamon	Supplemented with 75% oats + 5% cinnamon	
Moisture	3.17 ± 0.20^a^	3.52 ± 0.13^a^	3.32 ± 0.24^a^	3.07 ± 0.13^a^	1.19
Total lipids	16.78 ± 0.15^b^	17.76 ± 0.20^a^	17.84 ± 0.08^a^	18.65 ± 0.09^a^	0.92
Ash	1.83 ± 0.08^a^	1.96 ± 0.04^a^	2.15 ± 0.43^a^	2.65 ± 0.48^a^	2.13
Crud protein	5.38 ± 0.20^b^	7.50 ± 0.17^a^	7.65 ± 0.09^a^	8.52 ± 0.22^a^	1.15
Fiber	0.74 ± 0.16^b^	1.43 ± 0.25^b^	3.10 ± 0.20^a^	3.24 ± 0.20^a^	1.34
Total carbohydrate	72.38 ± 0.05^a^	67.83 ± 0.36^b^	65.93 ± 0.36^b^	63.88 ± 0.67^b^	3.08
Energy	455.62 ± 7.43^a^	461.18 ± 0.86^a^	454.91 ± 0.58^a^	457.46 ± 2.38^a^	25.71

Mean ± SD with the same latter in the same column are not significantly different (P ≤ 0.05) *: Least significant differences**.**

#### 3.1.3 DPPH radical scavenging activity

Table 3 illustrated that the ratio of antioxidant activity in both of whole grain oats and cinnamon. It's clearly seen from the results that, Whole grain oats had a high ratio of antioxidant activity which reached to 52.91%. Also cinnamon contains a high amount of antioxidant activity up to 87.91%.

**Table 3:** Antioxidant activity ratio by DPPH radical scavenging activity (%) in whole grain oats and cinnamon.

Component	Value
Whole grain oats	52.91%
Cinnamon	87.91%

### 3.2 Sensory evaluation of oats cookies treatments

Sensory evaluation of oats cookies treatments during storage at normal temperature for 3 months is noted in Table 4 .It is obvious from the result that appearance score of cookies treatments range between 8.35 and 8.59 at zero time. Also the results indicated that color results ranged from 7.98 to 8.64. Textures results in zero time are more highly acceptable and ranged from 8.34 to 8.65. Meanwhile taste score ranged between 7.97 and 8.89. Odor score ranged from 8.13 to 8.85. During the time of storage and after 3 months cookies treatment supplemented with 75% oats + 5% cinnamon recorded the best score, but control cookies treatment was the last. Ultimately, it could be inferred that all the cookies samples were acceptable for all the panelists relative to all sensory properties, i.e. appearance, color, texture, taste and odor. None the less, all properties of organoleptic evaluation significantly lowered (P ≤ 0.05) with the continued storage time proceeded, on the other hand all cookies treatments recorded a high score of sensory properties and also acceptable for human consumption, but cookies treatment supplemented with 75% oats + 5% cinnamon value was the highest of these properties.

**Table 4:** Organoleptic evaluation of cookies treatments.

Parameters	Time of Storage	Cookies treatments				L.S.D*_0.05_
		Control	Supplemented with 50% oats	Supplemented with 50% oats + 5% cinnamon	Supplemented with 75% oats + 5% cinnamon	
Appearance	Zero time	8.35 ± 0.75^a^	8.46 ± 0.56^a^	8.39 ± 0.82^a^	8.59 ± 1.13^a^	0.72
	After 1 month	7.41 ± 0.70^a^	7.44 ± 1.02^a^	7.45 ± 0.72^a^	7.49 ± 0.94^a^	0.78
	After 2 months	6.48 ± 0.41^a^	6.55 ± 0.68^a^	6.59 ± 0.61^a^	6.78 ± 0.41^a^	0.49
	After 3 months	5.32 ± 0.53^a^	5.46 ± 0.55^a^	5.75 ± 0.49^a^	5.88 ± 0.20^a^	0.43
Color	Zero time	7.98 ± 0.49^a^	8.39 ± 1.03^a^	8.45 ± 0.55^a^	8.64 ± 0.87^a^	0.69
	After 1 month	7.12 ± 0.51^a^	7.23 ± 0.64^a^	7.32 ± 0.51^a^	7.39 ± 0.56^a^	0.51
	After 2 months	6.34 ± 0.47^a^	6.54 ± 0.50^a^	6.55 ± 0.49^a^	6.69 ± 0.53^a^	0.45
	After 3 months	5.44 ± 0.49^a^	5.55 ± 0.47^a^	5.65 ± 0.72^a^	5.75 ± 0.49^a^	0.51
Texture	Zero time	8.34 ± 0.67^a^	8.45 ± 0.50^a^	8.55 ± 0.64^a^	8.65 ± 0.58^a^	0.54
	After 1 month	7.15 ± 0.58^a^	7.3 ± 0.82^a^	7.35 ± 0.69^a^	7.45 ± 0.59^a^	0.61
	After 2 months	6.1 ± 0.46^a^	6.35 ± 0.47^a^	6.45 ± 0.50^a^	6.55 ± 0.60^a^	0.47
	After 3 months	5.2 ± 0.66^b^	5.7 ± 0.48^a^	5.75 ± 0.42^a^	5.8 ± 0.63^a^	0.48
Taste	Zero time	7.97 ± 0.95^a^	8.35 ± 0.91^a^	8.85 ± 0.53^a^	8.89 ± 0.68^a^	0.72
	After 1 month	7.19 ± 0.36^a^	7.4 ± 0.74^a^	7.5 ± 0.47^a^	7.75 ± 0.42^a^	0.47
	After 2 months	6.38 ± 0.39^b^	7.05 ± 0.69^a^	7.2 ± 0.92^a^	7.45 ± 0.83^a^	0.67
	After 3 months	5.62 ± 0.61^a^	5.7 ± 0.63^a^	6.2 ± 0.59^a^	6.2 ± 0.59^a^	0.53
Odor	Zero time	8.13 ± 0.69^a^	8.45 ± 0.50^a^	8.65 ± 0.47^a^	8.85 ± 0.41^a^	0.48
	After 1 month	7.19 ± 0.49^b^	7.7 ± 0.48^a^	7.8 ± 0.42^a^	8.05 ± 0.72^a^	0.49
	After 2 months	6.51 ± 0.96^a^	6.8 ± 0.48^a^	7.05 ± 0.69^a^	7.15 ± 0.58^a^	0.64
	After 3 months	5.75 ± 0.79^a^	6.15 ± 0.47^a^	6.15 ± 0.78^a^	6.45 ± 0.69^a^	0.63

Mean ± SD with the same latter in the same column are not significantly different (P ≤ 0.05) *: Least significant differences.

### 3.3 Microbiological evaluation of oats cookies treatments

Table 5 illustrated the microbiological evaluation of cookies treatments during the time of storage period (3 months) in normal temperature. In zero time total bacterial counts results indicated that total bacterial counts score ranged from 0.82 × 10^2^ to 1.63 × 10^2^. It's obvious from the data that total spore forming bacteria count ranged from 0.1 × 10^2^ to 0.64 × 10^2^ cell/g at zero time. The change occurring in fungi and yeasts count (cfu/g) of cookies treatments were tabulated also in Table 5. It's clearly from the result that fungi and yeasts counts were ranged between 6.73 × 10^2^ and 8.65 × 10^2^ cell/g at zero time. During the subsequent storage period in all the cookies treatments were significantly increased, but cookies treatment supplemented with 75% oats + 5% cinnamon recorded the lowest number of total bacterial counts, spore forming bacteria and fungi and yeasts, on the other hand the value of control cookies treatment was the highest.

**Table 5:** Microbiological evaluation of cookies treatments.

Parameters	Time of Storage	Cookies treatments			
		Control	Supplemented with 50 % oats	Supplemented with 50% oats + 5% cinnamon	Supplemented with 75% oats + 5% cinnamon
Total bacterial counts	Zero time	1.63 × 10^2^	1.37 × 10^2^	0.91 × 10^2^	0.82 × 10^2^
	After 1 month	4.31 × 10^3^	3.7 × 10^3^	3.1 × 10^3^	2.9 × 10^3^
	After 2 months	7.91 × 10^3^	7.3 × 10^3^	6.8 × 10^3^	6.6 × 10^3^
	After 3 months	9.71 × 10^3^	9.4 × 10^3^	8.7 × 10^3^	8.49 × 10^3^
Spore forming bacteria	Zero time	0.64 × 10^2^	0.42 × 10^2^	0.18 × 10^2^	0.1 × 10^2^
	After 1 month	2.13 × 10^2^	1.81 × 10^2^	1.33 × 10^2^	1.23 × 10^2^
	After 2 months	4.25 × 10^2^	3.73 × 10^2^	3.02 × 10^2^	2.83 × 10^2^
	After 3 months	8.24 × 10^2^	6.62 × 10^2^	5.92 × 10^2^	5.73 × 10^2^
Fungi and yeasts	Zero time	8.65 × 10^2^	7.72 × 10^2^	6.93 × 10^2^	6.73 × 10^2^
	After 1 month	9.24 × 10^2^	8.33 × 10^2^	7.63 × 10^2^	7.50 × 10^2^
	After 2 months	9.84 × 10^2^	8.43 × 10^2^	7.93 × 10^2^	7.72 × 10^2^
	After 3 months	10.34 × 10^2^	9.43 × 10^2^	8.32 × 10^2^	8.13 × 10^2^

### 3.4 Biochemical assays in blood serum

#### 3.4.1 Effect of feeding with cookies treatments for 30 days on blood serum enzymes

Result of GPT, GOT, total cholesterol, triglyceride, HDL, LDL, urea, creatinine and glucose are presented in table 6.

**Table 6:** Effect of feeding cookies treatments for 30 days on biological assays enzymes for male albino rats.

Parameters	Groups				L.S.D*_0.05_
	G1	G2	G3	G4	
GPT(U/L)	22.00 ± 1.00^c^	205.67 ± 1.53^a^	208.33 ± 2.65^a^	27.00 ± 2.65^b^	3.92
GOT (U/L)	24.67 ± 2.08^c^	269.00 ± 2.00^a^	260.67 ± 2.00^b^	26.67 ± 3.21^c^	4.51
Cholesterol (mg/dl)	75.03 ± 1.58^b^	198.8 ± 3.64^a^	197.73 ± 2.33^a^	66.33 ± 2.47^c^	4.92
Triglyceride (mg/dl)	86.06 ± 1.23^b^	276.00 ± 2.09^a^	275. 5 ± 4.71^a^	88.67 ± 2.91^b^	5.79
HDL	57.00 ± 1.00^c^	87.00 ± 1.00^a^	83.00 ± 2.00^b^	53.67 ± 1.53^d^	2.72
LDL	52.67 ± 3.21^b^	405.67 ± 1.53^a^	403.67 ± 2. 52^a^	50.67 ± 1.53^b^	4.35
Urea (mg/dl)	20.3 ± 1.2^c^	84.07 ± 3.07^a^	80.4 ± 1.01^b^	18.50 ± 0.89^c^	3.98
Creatinin (mg/dl)	0.71 ± 0.04^b^	1.72 ± 0.03^a^	1.73 ± 0.02^a^	0.65 ± 0.02^c^	0.05
Glucose	98.33 ± 3.06^d^	307.00 ± 3.61^a^	293.67 ± 4.04^b^	114.67 ± 4.51^c^	7.23

Mean ± SD with the same latter in the same column are not significantly different (P ≤ 0.05).

*: Least significant differences.

Group 1: Control negative group (−ve), in which normal rats were fed on basal diet and tap water.

Group 2: Control positive group (+ve), in which rats inflicted with hepatotoxicity by CCl_4_were fed on basal diet and tap water.

Group 3: Rats with impaired liver, treated with cookies without oats.

Group 4: Rats with impaired liver treated with oats cookies (prepared with 75% oats and 5% cinnamon).

### 3.5 Alanine amino transferase (ALT) enzyme

Data presented in Table 6 revealed the effect of feeding rats with tested materials on the serum level of alanine amino transferase (ALT) enzyme. It could be noticed from the present table that rats without treatment, positive group (C + ve) the mean value of ALT enzyme was 205.67 ± 1.53 U/L while the normal rats (C-ve) group was 22.00 ± 1.00 U/L and showed a significant differences at (p ≤ 0.05). In the G4 we found a big improvement in ALT enzyme but G3 which rats were fed on cookies diet without oats did not have positive effect.

### 3.6 Aspartate amino transaminase (AST) enzyme

Data presented in the same Table 6 revealed the without treatment (C + ve), indicated enzyme activity as 269.00 ± 2.00 U/L while negative group was 24.67 ± 2.08 U/L. For rats fed on treatments G4 it could be noticed that the major value of serum ALT of these significant differences when compared to (C + ve) group and G3.

### 3.7 Total cholesterol (TC)

Data presented in table 6 showed mean value ± SD of serum cholesterol (C + ve) the group was 198.8 ± 3.64 mg/dl compared to 75.03 ± 1.58 mg/dl in normal group. The mean value of rats fed on diets of G4 that was showing significant decreases as compared to positive group, but G3 didn't show any significant difference.

### 3.8 Triglycerides (TG)

Concerning triglycerides, the data in the same Table 6 revealed that the mean value of rats (C + ve) group, serum triglycerides was 276.00 ± 2.09 mg/dl as compared to normal rats which were 86.06 ± 1.23 mg/dl. It was illustrated a significant increase in serum triglycerides levels in the positive group, when compared to the normal rats. For rats fed on treatments in G4 it could be concluded that the serum triglycerides mean value was 88.67 ± 2.91 mg/dl, respectively and showing significant difference, as compared to (C + ve) group. There is no significant variance between G3 and positive control group.

### 3.9 High density lipoprotein cholesterol (HDL-c)

Data presented in Table 6 showed the effect of feeding with tested materials on serum levels of (HDL-c). It is obvious that rats in (C + ve) group, the mean value of serum HDL-c level were 87.00 ± 1.00 mg/dl. In normal rats the mean value of the serum HDL-c level was 57.00 ± 1.00 mg/dl. This result indicates that there was a significant increase in HDL-c in the serum (C + ve) group as compared to (C-ve). All mean values of serum HDL-c of G4 was lower than the negative positive group and being showing a significant difference as compared to (C + ve) group on the other hand G3 being showing no significant difference as compared to (C + ve) group.

### 3.10 Low density lipoprotein cholesterol (LDL-c)

Data presented in Table 6 showed the serum levels of (LDL-c) were significant elevated in the positive group to 405.67 ± 1.53 mg/dl from 52.67 ± 3.21 mg/dl in the normal group. Rats in G4 showed a significantly decrease in the previously mentioned parameter as compared to (C + ve) group. Meanwhile G3 showing no significant difference as compared to (C + ve) group.

### 3.11 Serum urea

Data in Table 6 showed the effect of feeding rats with tested materials on serum urea (mg/dl). The mean value of serum urea for the experimental group of feeding on basal diet only was 20.3 ± 1.2 mg/dl while urea for the positive control group was 84.07 ± 3.07 mg/dl. Results revealed that, rats group (C + ve) have a significant increase (P ≤ 0.05) in serum urea when compared with normal group. The mean value of urea of G4 was showing a significant decrease as compared to the positive group.

### 3.12 Serum creatinine

Data presented in the same Table 6 showed the effect of feeding rats oats cookies on creatinine (mg/dl). It could be noticed that the serum creatinine of rats fed on high diet without treatment was 1.72 ± 0.03 mg/dl while normal rats group (C-ve) was 0.71 ± 0.04 mg/dl which showing a significant decreasing as compared to negative control group. When the rats fed on G4 occurred significant differences in serum creatinine levels as compared to the positive control group except for G3. A lot of animal's studies proved that intake oats have favorable effects on the function of kidney. But these effects haven't been assessed in humans who consumption oats.

### 3.13 Glucose levels

Data in Table 6 presented the effect of feeding with tested materials on glucose levels. It could be concluded that, the mean value ± SD of glucose of (C + ve) group significantly increased as compared to normal rats, it was being 307.00 ± 3.61 mg/dl and 98.33 ± 3.06 mg/dl, respectively. In G4 there was a significant increase in the levels of glucose as compared to normal group and a significant decrease as compared to positive group. Finally G3 (rats which were fed on diet contained 0% oats) didn't show any significant difference in glucose level.

### 3.14 Histopathological examination

#### 3.14.1 Histopathological examination of liver

**Fig. 1:**
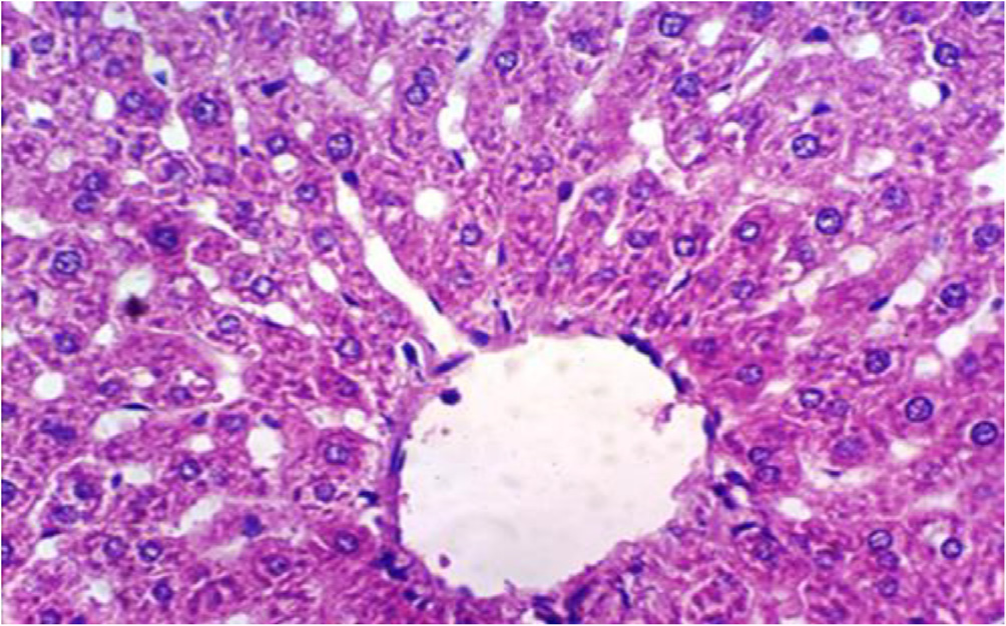
Histopathological examination of liver cells of rat from group 1 showing the normal histological structure of hepatic lobule (H & E X 400).

**Fig. 2:**
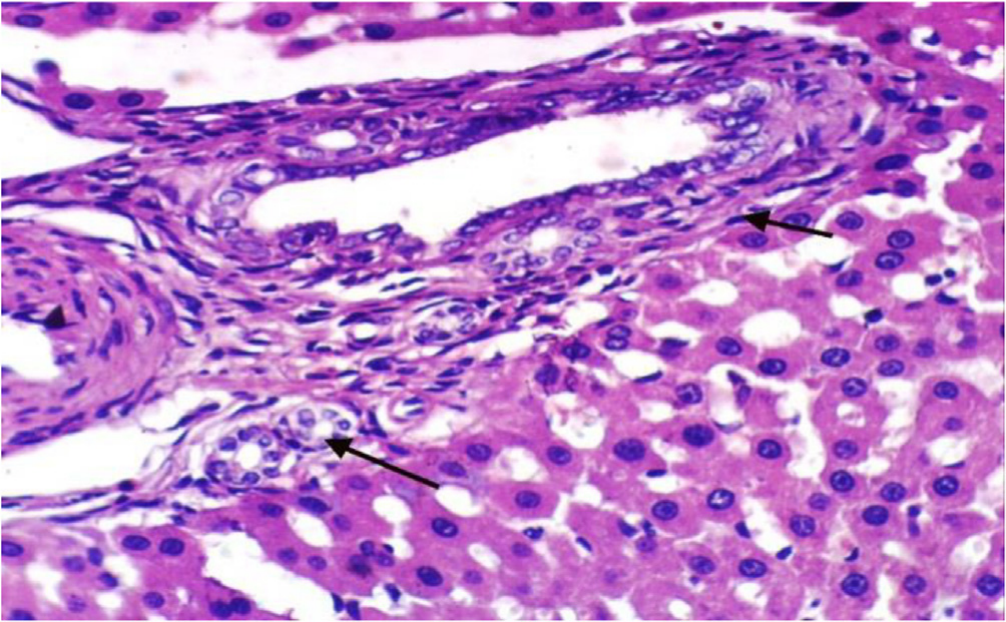
Histopathological examination of liver cells of rat from group 2 showing fibroblasts proliferation in the portal triad and appearance of newly formed bile ductules and focal hepatocellular necrosis associated with inflammatory cells infiltration (H & E X 400).

**Fig. 3:**
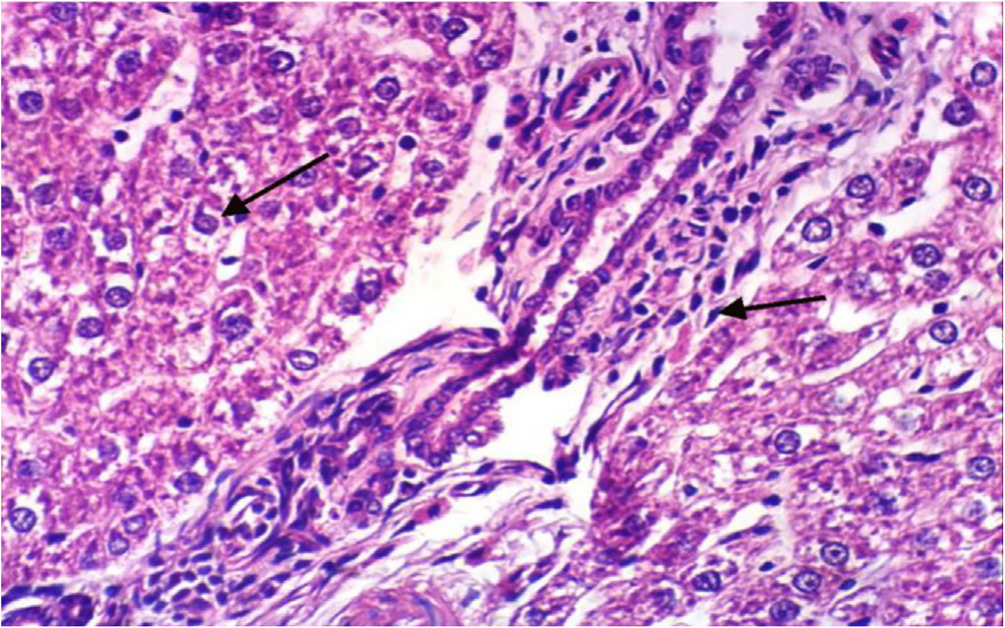
Histopathological examination of liver cells of rat from group 3 showing hydropic degeneration of hepatocytes and portal infiltration with mononuclear cells (H & E X 400).

**Fig. 4:**
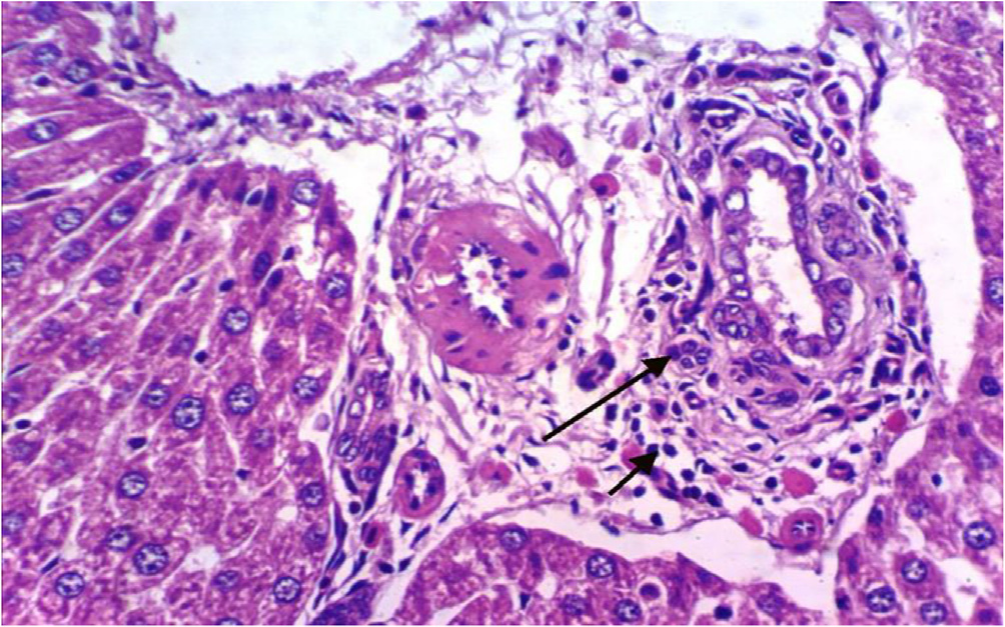
Histopathological examination of liver cells of rat from group 4 showing slight fibroplasia in the portal triad, appearance of newly formed bile ductules and slight hydropic degeneration of some hepatocytes (H & E X 400).

Liver of rats from negative group 1 revealed the normal histological structure of hepatic lobule Fig. 1. On the contrary, the liver of rats from group 2 showed fibroblasts proliferation in the portal triad, appearance of newly formed bile ductules, focal hepatocellular necrosis associated with inflammatory cells infiltration and fibrosis of the hepatic capsule Fig. 2. Moreover, the liver from group 3 revealed hydropic degeneration of hepatocytes Fig. 3. Examined sections from group 4 showed slight hydropic degeneration of some hepatocytes, slight fibroplasia in the portal triad and appearance of newly formed bile ductules Fig. 4.

#### 3.14.2 Histopathological examination of kidneys

**Fig. 5:**
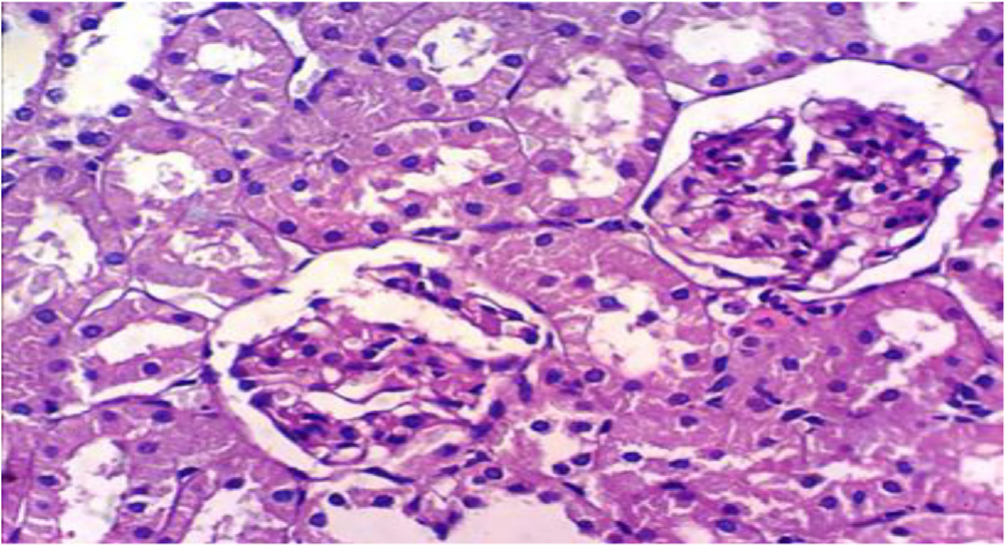
Photomicrograph of kidney of rat from group 1 showing the normal histological structure of renal parenchyma (H & E X 400).

**Fig. 6:**
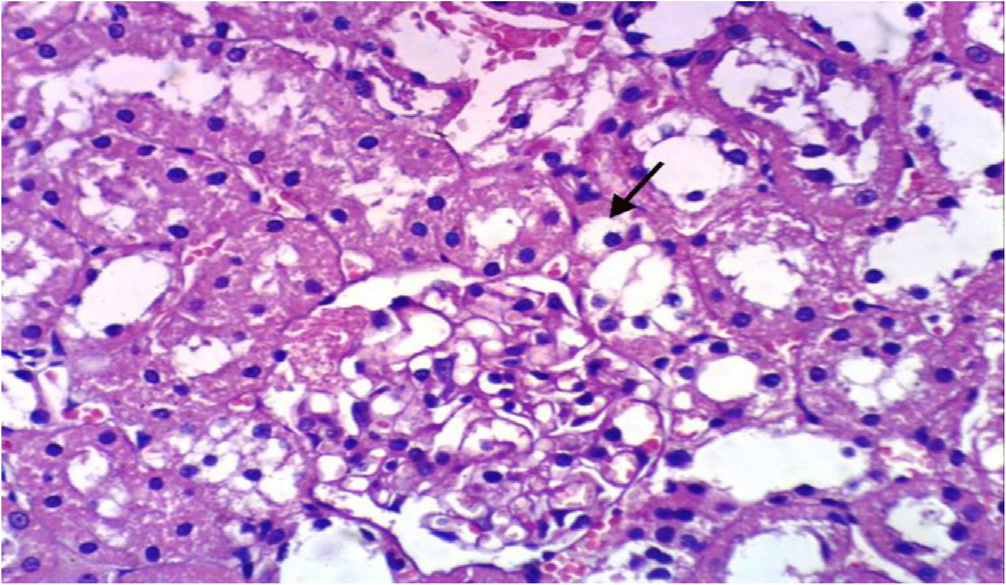
Photomicrograph of kidney of rat from group 2 showing vacuolation of renal tubular epithelium and proteinaceous material in the lumen of renal tubules (H & E X 400).

**Fig. 7:**
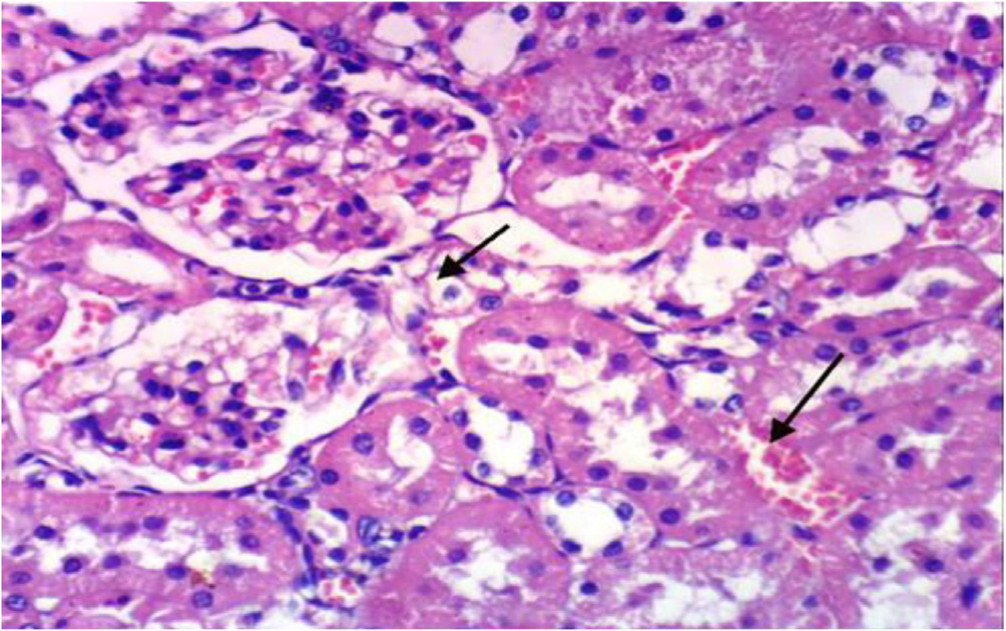
photomicrograph of kidney of rat from group 3 showing proteinaceous material in the lumen of renal tubules and congestion of intertubular blood vessels (H & E X 400).

**Fig. 8:**
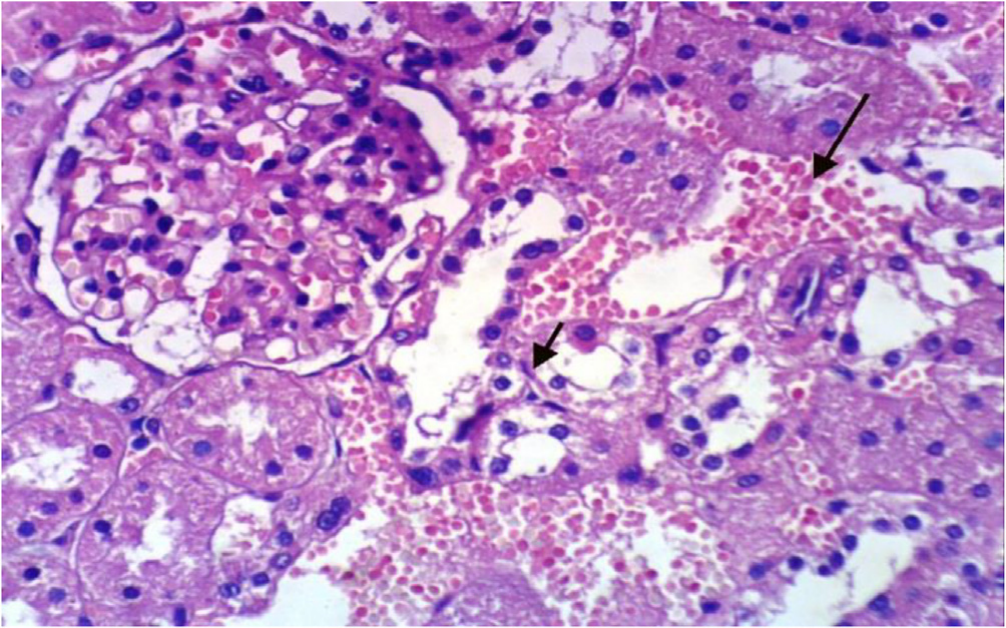
photomicrograph of kidney of rat from group 4 showing vacuolation of epithelial lining some renal tubules and focal renal haemorrhage (H & E X 400).

Microscopically, kidneys of rats from group 1 revealed the normal histological structure of renal parenchyma Fig. 5. On contrary, kidneys of rats from group 2 showed proteinaceous material in the lumen of renal tubules Fig. 6. Moreover, liver of rats from group 3 revealed proteinaceous materials in the lumen of renal tubules, congestion of intertubular blood vessels and focal necrosis of renal tubules associated with inflammatory cells infiltration Fig. 7. On the other hand, kidneys from group 4 showed proteinaceous material in the lumen of some renal tubules, slight vacuolation of epithelial lining some renal tubules and slight congestion of intertubular blood capillaries Fig. 8.

## 4 Dissection

Oats are a type of cereal crop. In recent years it has attracted a lot of attention because of its high nutritional value**.** So that oats have an important role in the food industry. Cinnamon is also used widely in industry as a flavoring agent and spice. The results of oats chemical composition are agreement with **(**Petkov et al., 2001) who said that oats grain contain more high amount of protein than the other cereals, however lysine as yet the limiting amino acid. Similarly, (Sibakov et al., 2013) noted that oats flour have great health benefits and that may be due to its composition. In addition, it has been shown that the oats flour chemical composition was (5.9%) fat and (13%) protein. Fulgoni et al., 2015 noted that oats contain dietary fiber, vitamins as well as minerals. Also (Pomeranz, 1982) said that oats contain on a high contents of protein and oil compared to the other cereal grains.

Also the antioxidant result of oats agreement with Collins et al., 1991, Peterson et al., 2001 who reported that oats considered one of the most common whole grain cereal as it contain many types of phytochemicals like phenolic acids, avonoids, sterols, tocotrienols as well as phytic acid have a high amount of phenolic antioxidants and avenanthramides. Bei et al., 2017 reported that phenolic compounds of oats are considered as an important natural source of antioxidants properties. The cinnamon antioxidant result agreed with Morais et al., 2009 who said that antioxidants are compounds that can prevent or delay (nonenzymatic or enzymatic) oxidation. Cinnamon oil (*Cinnamomum cassia*) contains phenolic compounds such as cinnamyl acetate, eucalyptol and cinnamic aldehyde which have antioxidant effects. Sá et al., 2015 said that cinnamon is considered the most important flavored spices in the food industry as its extracts can be used as antioxidant activities. The oats cookies chemical composition results are in conformity with Flander et al., 2007, Yao et al., 2007 who reported that beta glucan of oats have many advantages of the unique properties in the food industry for the production of new food products by incorporating oats beta glucan into various food products including: cookies, beverages, bread, infant foods and breakfast. Recently oats cookies become the favorite snack and that due to its high nutritional value and low price. Verardo et al. (2011) said that oats products aren't only contain a high amount of functional molecules like proteins, peptides, amino acids, vitamins and dietary fibers but it also has a phytochemicals such as lignans, polyphenols, and phenolic acids and phytate that are concentrated in the outer layers of the grain.

The sensory evaluation of oats cookies results are agreed with Swapna & Rao, 2016 who said that cookies supplemented with oat by 25% acceptable for its organoleptic properties as the results obtained showed a high score especially overall acceptability. Duta & Culetu, 2015 concluded that flavor directly proportional to levels of oats as increasing in the levels of oats make flavor stronger. Francis & Clydesdale, 1975 studied the differences in color between cookies made from white flour and that supplemented with oats by the human eye. And they found in the case of addition oat by 30%, 50% and 70% the differences in color are not obvious by the human eye but the addition oat by 100% they noticed the difference is visible to the human eye. Sindhi et al., 2013 demonstrated that oats antioxidants contain many important industrial as it retaining the texture, taste, flavor, slowing the lipid oxidation in addition to that their nutritional content.

The microbial evaluation all cookies treatment during subsequent the period of storage were in the permissible limits as recommended by (Egyptian Standard Specification (ESS), No, 416–2, 2008). Also the results obtained are consistent with Ayaz et al., 2008 who said that phenolic extracted from oats proofer to prevent B. subtilis from growth. Katalinic et al., 2010 concluded that phenolic compounds of oats have anti-microbial properties. Zhang et al., 2020 reported that avenanthramides of oats positively affect the intestinal microflora, as well as decrease the pathogenic bacteria. Sadeghi et al., 2019 said that cinnamon one of natural component, which contains many pharmacological functions such as anti-microbial. Vasconcelos, et al., 2018 found that leaves and barks of cinnamon have an anti-bacteria and anti-fungal properties.

The same conclusion was mentioned by Sreelatha et al., 2009 who studied the impact of EA fraction extracted from oats on the liver damage induced by alcohol, and they found a decreases in the levels of liver enzymes and that may be due to the hepatic tissue damage was repaired and stabilize plasma membrane induced by alcohol. In addition to that used EA fraction in treated significantly prevent pathological from liver degeneration changes (macrovesicular and microvesicular), which happened by alcohol in the liver cells as well as retention of the cellular integrity of the liver cells. Hassanein & El-Amir, 2017 found that pre-treatment with EA fraction, which contain avenanthramides made decreases in the levels of liver enzymes in blood serum, in addition to facilitate the prevention of alcoholic liver injury. Hager et al., 2013 concluded that too much fiber help the liver to work well. Oats can helps get rid of some extra belly fat and are a good way to prevent liver disease. Generally the increases in consumption of oats products have beneficial impacts population. As it makes a decrease in the severity, incidence of atherosclerosis, in addition to its ability to decrease lipid percentage (Kerckhoffs et al., 2002, Kelly et al., 2007). Intake oats supplementation every day, have relationship with reducing the risk of gastrointestinal disorders, and cardiovascular disease (Mir et al., 2018). Andersson et al., 2010 mentioned that in the Western oats supplemented diet make a decrease in plasma cholesterol, lower some inflammatory markers levels, also inhibits the development of an atherosclerotic lesion in LDL in rats. Liu et al., 2011 studied the effects of oats antioxidant (AVA) on human serum by measured antioxidant status and plasma lipid peroxides. They found a decrease in the level of malondialdehyde (MDA) (significantly lowered by 28.1%) and the levels of triglyceride (TG), low density lipoprotein cholesterol (LDL-C) and the total cholesterol (TC) were significantly decreased by 28.1, 15.1 and11.1 %, respectively. In the other hand, they reported an increase in the levels of glutathione hormone (GSH) and serum superoxide dismutase (SOD) by 17.9 and 8.4 respectively. In addition, the levels of high density blood lipoprotein cholesterol (HDL-C) were increased by 13.2%. Rouhani et al., 2018 studied the effect of intake oats on biomarkers of renal function in patients with chronic kidney disease (CKD). 52 patients with CKD were at random assigned to a control group (recommended to decrease intake potassium, phosphorus, sodium and dietary protein) or an oats consumption group (given nutritional recommendations for controls 50 g oats /day). Urine protein, blood urea nitrogen (BUN), serum creatinine (SCr), urine creatinine, serum albumin, serum potassium, parathyroid hormone (PTH), and serum klotho concentration were measured at baseline and after an 8 weeks intervention, so oats has many beneficial effects on serum potassium and serum albumin in patients with CKD. Tapola et al., 2005, Zerm et al., 2013 oats flour were more effective against two kinds of diabetes than oats bran crisp and that due to beta glucan content in oats were higher by 3 times than oats bran crisp. After intake the oats whether crisp or bran flour, the blood glucose levels were decreased at 15, 30, 45 min but at 90 min become higher after 12.5 g glucose loading. Thus, beta glucan reduce appetite and decrease food intake. Oatmeal diets every day can improve resistance of insulin in the patients with type 2 of diabetes. Gazal et al., 2014 said that intake oats can make a lower in the blood sugar through a long period of time.

## 5 Conclusions

This study demonstrated that oats contain high nutrition value compared to other grains so in our study we supplemented cookies with oats and cinnamon to increasing the nutritional value of the product, dietary fiber as well as improve its sensory properties. Oats also content of high antioxidant activity that led to reduced microbial growth significantly in the product. And we have provided evidence that oats a significant effect on biochemical assays in blood serum in rats with cirrhosis by CCL4. We found a decreased in liver and kidney enzymes, HDL, LDL, cholesterol, triglyceride and blood glucose. In addition to slight improvement in damaged liver and kidney tissues by carbon tetrachloride CCL4. Finally, the high nutritional value of oats should be brought to the public attention through product branding, targeted communication and advertising. Also there is a need to innovation many products supplemented with oats that going hand in hand with fill the shortage of knowledge about the effect of processing on health values and nutritive.

## Declaration of Competing Interest

The authors declare that they have no known competing financial interests or personal relationships that could have appeared to influence the work reported in this paper.
